# Supplementary material for: Charge transfer to ground-state ions produces free electrons
Source: Nat Commun. 2017 Jan 30;8:14277. doi: 10.1038/ncomms14277 (PMC5290264; doi:10.1038/ncomms14277)
Supplement: Supplementary Information — Supplementary Figures, Supplementary Tables, Supplementary Notes, and Supplementary References [file ncomms14277-s1.pdf]

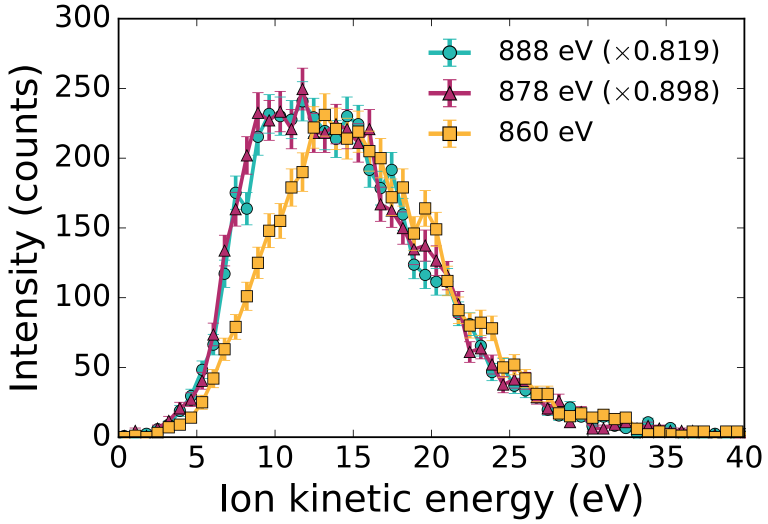

Supplementary Figure 1 | Number of events vs. total ion kinetic energy (KER). The data measured at 878 and 888 eV were divided by factors of 1.114 and 1.221 (see Supplementary Note 4).

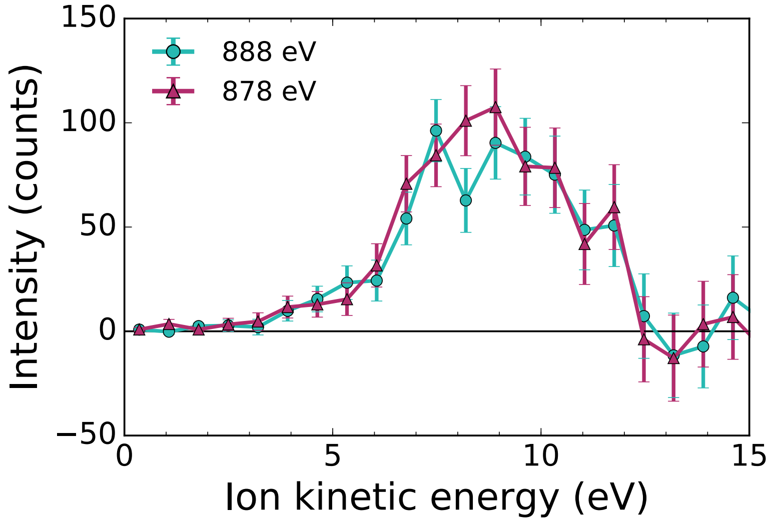

Supplementary Figure 2 | Number of events vs. total ion kinetic energy (KER). A background unrelated to Ne photoionization has been subtracted (see Supplementary Note4).

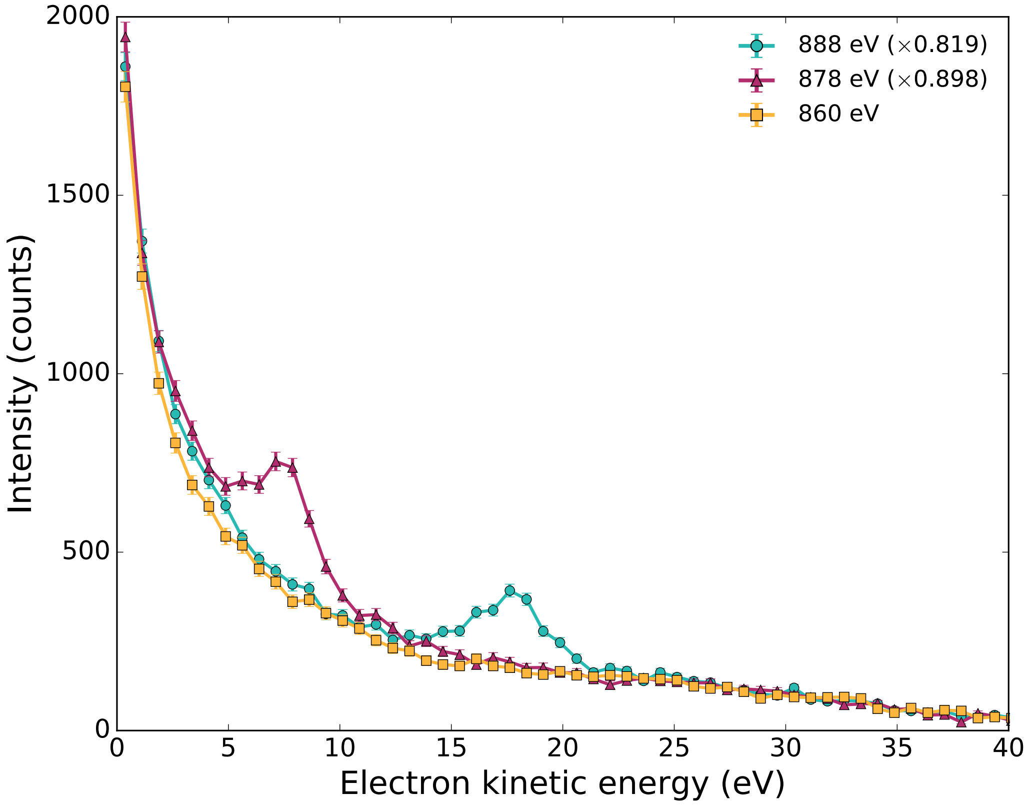

Supplementary Figure 3 | Electron spectra in coincidence with a Ne^+ and two Kr^+ fragments for a wide electron kinetic energy range. The spectra measured at 888 and 878 eV were divided by factors of 1.114 and 1.221 to match the intensity of the background spectrum in the 25–40 eV kinetic energy interval (see Supplementary Note 4).

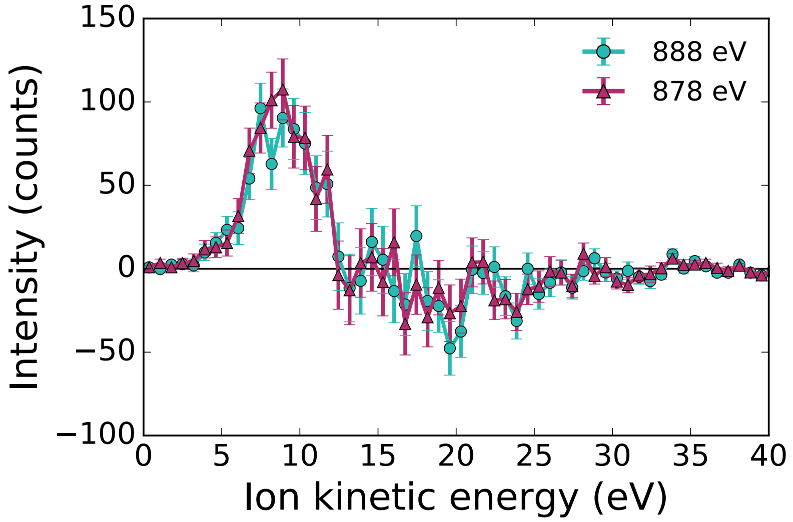

Supplementary Figure 4 | Number of events vs. total ion kinetic energy (KER). Background events were subtracted after scaling by procedure 1. (A subset of these data is shown in Supplementary Fig. 2.)

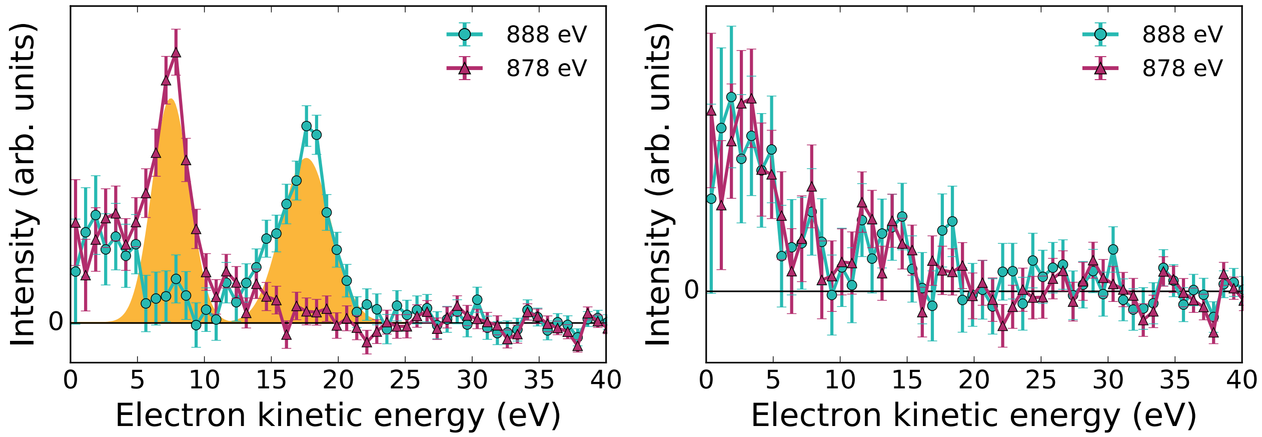

Supplementary Figure 5 | Electron spectra in coincidence with a Ne^{+} and two Kr^{+} fragments for a wide electron kinetic energy range. Background events were subtracted after scaling by procedure 1. Left panel: Spectra showing all background subtracted electrons together with the profiles used to assess the contributions of Ne 1s photoionization, right panel: Spectra after subtraction of the Ne 1s component.

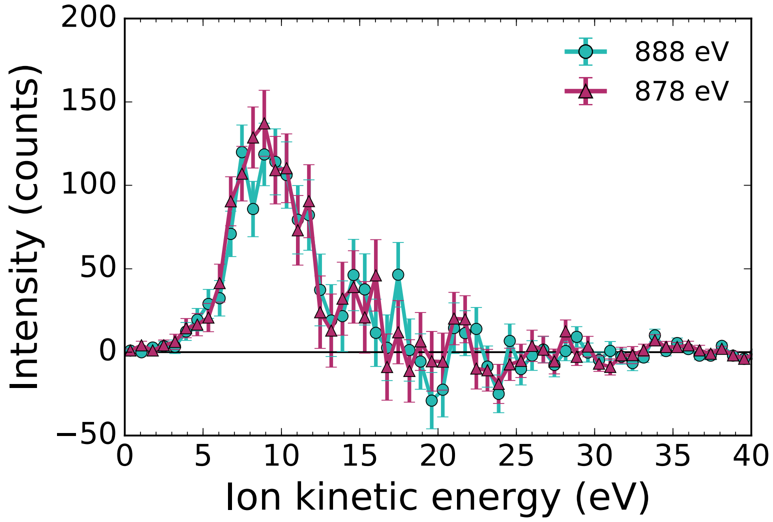

Supplementary Figure 6 | Number of events vs. total ion kinetic energy (KER). Background events were subtracted after scaling by procedure 2.

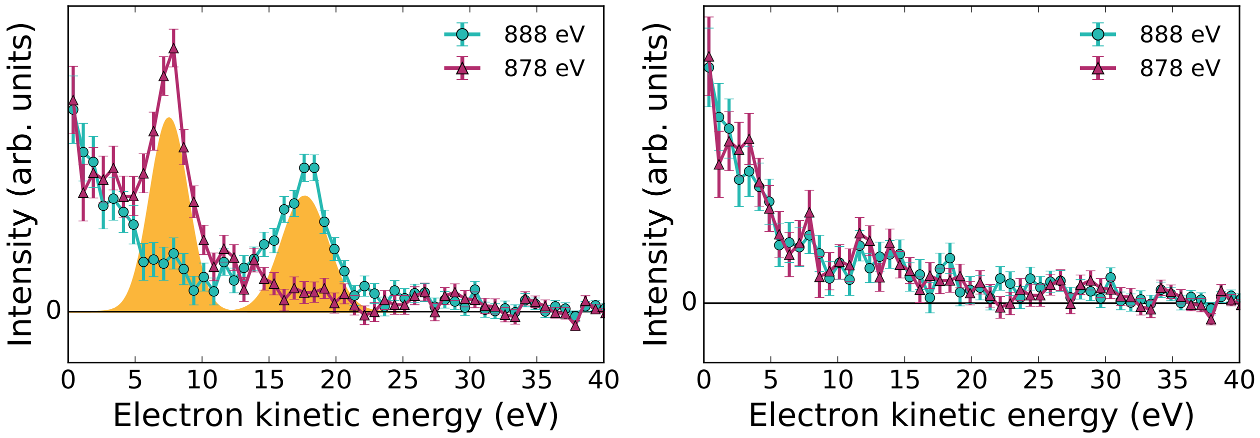

Supplementary Figure 7 | Electron spectra in coincidence with a Ne^{+} and two Kr^{+} fragments for a wide electron kinetic energy range. Background events were subtracted after scaling by procedure 2. Left panel: Spectra showing all background subtracted electrons together with the profiles used to assess the contributions of Ne 1s photoionization, right panel: Spectra after subtraction of the Ne 1s component. The two spectra in the right hand side panel were further normalized by the respective cross sections for Ne 1s photoionization.

Supplementary Table 1 | Factors for background subtraction procedure 1.

	**888 eV**	**878 eV**	**860 eV**
Factor A	1.221	1.114	1.000

Spectra are divided by the respective factors.

Supplementary Table 2 | Electron relative populations in procedure 1.

Measured		Estimated	
Photoelectron	1.00	Photoelectron	1.000
0–11 eV	0.73 ± 0.09	ETMD or ICD (Low)	1.163
11–20 eV	0.25 ± 0.05	ICD (High)	0.194
20–25 eV	0.00 ± 0.03		
Sum (exclude photoelectron)	0.97	Sum (exclude photoelectron)	1.357

Supplementary Table 3 | Factors in procedure 2.

	888 eV	878 eV	860 eV	Note
(a) Time (hours)	4.936	4.263	3.916	
(b) Current from light intensity monitor (nA)	2.381	2.506	2.745	Au thin film was used for the monitor.
(c) Photoabsorption cross section of Au (Mb)	2.024	2.069	2.150	Interpolation of data in Supplementary Ref. 1.
(d) Relative intensity of the incident light	1.176	1.211	1.277	= (b) / (c)
(e) Photoabsorption cross section of Kr (Mb)	0.543	0.559	0.587	Interpolation of data in Supplementary Ref. 2.
(f) Time × Light Int. × Kr cross section	3.155	2.886	2.933	= (a) × (d) × (e)
(g) Factor A	1.076	0.984	1.000	Proportional to (f)

Supplementary Table 4 | Electron relative populations in procedure 2.

Measured		Estimated	
Photoelectron	1.00	Photoelectron	1.000
0–11 eV	1.57 ± 0.17	ETMD or ICD (Low)	1.163
11–20 eV	0.46 ± 0.06	ICD (High)	0.194
20–25 eV	0.09 ± 0.02		
Sum (exclude photoelectron)	2.12	Sum (exclude photoelectron)	1.357

Supplementary Note 1. Event sets

Our analysis of cluster radiationless deexcitation is based on detection of the cluster ionic fragments together with at least one electron in a multi-particle coincidence. In the ionic fragments, the signature of the ETMD process we demonstrate here is seen as the coincident detection of one Ne^+ and two Kr^+ fragments (see Fig. 1 of the main manuscript). The respective ionic fragments can be identified by their time-of-flight (TOF), as shown in Fig. 2b of the main manuscript. We have therefore selected in our data set all events in which at least one Ne^+ fragment, two Kr^+ fragments and one electron were received. All Kr^+ fragments emitted into 4π sr were collected. Since some Ne^+ emitted into the detector direction overlapped with Ne^{2+}, we used only Ne^+ emitted backward (2π sr) for ion kinetic energy analysis. We have thus arrived at three set of events, recorded at 860 eV, 878 eV and 888 eV of photon energy, from which all electron spectra shown in the main text and all electron and ion spectra in this Supplementary Information were derived. Referring to the Fig. 2b of ion coincidences in the main text, all data discussed here (and in further Figures of the main text) pertain to the TOF combinations highlighted there.

From Fig. 2b of the main text, it can be seen that the signature of one Ne^+ fragment, two Kr^+ fragments is more abundant than other combinations of three ions. We have not attempted to analyze the competition of this channel with channels producing only two ions, though. Due to the presence of the ETMD channels we think it is unlikely that the former have more than a marginal branching ratio, though. The only such mechanism we can think of is radiative charge transfer (RCT), in which a Ne^{2+} produced in Auger decay would transfer one positive charge to a neighbor while emitting a photon, thus resulting in $2Ne^+$. This channel has been seen after L-shell Auger decay in Ar clusters, but only from states for which all non-radiative decay channels are closed. This is because the RCT decay rate is much slower (at least two orders of magnitude) than ETMD and ICD[3,4]. A recent study of a mixed rare gas system that experimentally showed the dominance of ETMD over radiative decay channels is in Supplementary Ref. 5. Radiative charge transfer might become important in clusters that contain only a single Kr atom in total. Due to the stochastic nature of cluster formation and the rather small mean size of our clusters, those might be present in our jet. We have not made an attempt to search for their decay signature.

Supplementary Note 2. Ion spectra

In Fig. 2 of the main text, we depict the TOF spectrum of the ion manifold. It has been shown in several studies, that important information on ultrafast radiationless relaxation processes in clusters can be obtained by plotting the ion intensities vs. their kinetic energies, or vs. the total kinetic energy imparted to the ions ('kinetic energy release', KER). The latter information is shown in Supplementary Fig. 1.

Supplementary Fig. 1 clearly shows the presence of an additional component in the KER spectrum as soon as the energy is tuned above the Ne 1s threshold. Compared to the events present already below energies at which Ne core ionization is possible, their KER as rather low. The nature of these background events is not known to us presently, and has to be clarified in future experiments. A comparison of the intensities between the Ne-related contribution and the background is therefore not meaningful in our opinion.

In order to allow for a better assessment of the Ne-related component in KER spectrum, we show it after background subtraction in Supplementary Fig. 2. If we assume the equilibrium geometry of the $NeKr_2$ trimer reported by Stumpf *et al*.[6] (KrNeKr angle θ = 67.01 degree and Ne-Kr interatomic distance r = 3.68 Å), the sum of the energies of three ions (Ne^+ and two Kr^+) is 10.08 eV, which is in reasonable agreement with the high kinetic energy flank of the peak in the spectrum. The plot extends towards lower total ion energies ('kinetic energy release', KER) and the peak is broad, because our clusters were on average larger than a trimer and neutrals fragments take some momentum.

Supplementary Note 3. Electron spectra and background subtraction

In order to isolate the kinetic energy of electrons resulting from ETMD after Ne 1s Auger decay, we have to subtract from the raw electron spectra the contributions of events driven by photoionization of the Kr atoms in the clusters, and the contributions of the Ne 1s photoelectrons. As explained in the main text, we do this in two steps: Firstly, we subtract the Kr-related contributions to the spectrum by estimating them from a spectrum measured below the Ne 1s ionization threshold, and secondly we subtract the Ne 1s photoline by fitting it using a Gaussian profile to it.

As the spectra above and below the Ne 1s threshold were acquired independently, some scaling of the Kr background spectrum is inevitable for a correct subtraction. In Supplementary Fig. 3 we show spectra before background subtraction, but already with the scaling factors of the procedure adopted in the main text applied. Consequences of an alternate choice of the background scaling factor are described and compared in the following Supplementary Note 4.

The main point in Supplementary Fig. 3 is the presence of a difference between the yellow background curve and the two other traces, which is outside of the respective error bars for kinetic energies up to 20 eV.

After subtraction of the background unrelated to core level photoemission of Ne, we arrive at spectra containing the slow electrons collected in the process. These consist of the Ne 1s core level photoelectrons, those can clearly be seen at approx. 8 and 18 eV of kinetic energy, and the electrons from radiationless decay, most importantly ETMD after Auger decay (Fig. 3f of the main text). In order to assess the intensity arising from ETMD, the Ne 1s component is subtracted as well. This step of data treatment is illustrated in Fig. 3f and 3g of the main text, and for a wider energy range in Supplementary Fig. 5.

Supplementary Note 4. Background scaling

As explained in the preceding section, we have subtracted the background unrelated to Ne 1s photoionization using a pragmatic approach, which compared the intensity in a spectral region in which no Ne-related features are expected ('Procedure 1'). Alternatively, if all of the background arises from Kr photoionization, it should be possible to relate the intensity of the background above and below the Ne photoionization threshold based on the ratio of the pertaining cross sections (for photoionization of Kr). In this approach ('Procedure 2'), also other wavelength dependent factors, such as the spectral dependence of the synchrotron radiation intensity, have to be taken into account. In order to assess the robustness of our results, in the following we compare results of the two different methods for background subtraction.

The difference between the two methods essentially boils down to the normalization factors applied to the 878 eV and 888 eV spectra before subtraction of the 860 eV spectrum, which in the following is called

‘Factor A’.

In order to allow the reader to appreciate the difference between both methods, we present the difference spectra for the ion intensities vs. total KER (before background subtraction in Supplementary Fig. 1), for the electron intensities vs. electron kinetic energy (before background subtraction in Supplementary Fig. 3), and the results for the normalized electron populations (Table 1 of the main text), each in two different versions.

Procedure 1 (applied in publication). We determine Factor A such that the sum of counts in the subtracted electron spectrum in the kinetic energy interval 25–40 eV is zero, i.e. [888 eV or 878 eV spectrum] / Factor A – [860 eV spectrum]. The actual values are listed in Supplementary Table 1. Ion intensities vs. total ion kinetic energy and electron intensities vs. electron kinetic energy are plotted in Supplementary Figs. 4 and 5, respectively. The normalized electron populations are listed in Supplementary Table 2.

Procedure 2 (variant). Alternatively, we determine Factor A according to the relative intensities obtained by taking into account the respective cross sections for Kr photoionization (believed to be responsible for the background), the total acquisition times and the integrated values for the synchrotron radiation flux. These factors are collected in Supplementary Table 3. Ion intensities vs. total ion kinetic energy and electron intensities vs. electron kinetic energy are plotted in Supplementary Figs. 6 and 7, respectively. The normalized electron populations are listed in Supplementary Table 4.

The electron spectra, shown in Supplementary Figs. 4 and 6, give no hint which of the methods is preferable. The ion difference spectra to which scaling procedure 1 was applied however display some data points with negative intensity, for total ion energies of 18–20 eV. This is an indication that the background intensity is somewhat overestimated by this method. Intensity in the respective region is around zero for procedure 2. Clearly, subtraction of the 860 eV spectrum rests on the assumption that the mechanism of background production is not altered as a function of photon energy. We have not been able to develop a model for production of the background counts, therefore currently we cannot assess this assumption, and refrain from further discriminating between the two models. For the presentation of the main manuscript we have decided to stick to the more conservative numbers from procedure 1.

With respect to the comparison between experimental and estimated theoretical results for the low kinetic energy electron intensities, values from procedure 1 are slightly lower than theory, while values from procedure 2 are above the theoretical estimates. In principle, inelastic scattering of Auger or valence photoelectrons could lead to the production of additional low kinetic energy electrons that appear only in the experimental balance, but for this experiment we have no evidence for contributions from this channel (see next Supplementary Note 5).

Supplementary Note 5. Influence of inelastic scattering

We would briefly like to discuss the potential influence of inelastic collisions on our results. Intercluster inelastic collisions usually are unimportant for cluster beams in the density range used in our experiments, we therefore focus on intracluster inelastic collisions. Here, two different channels have to be discussed:

1. Losses of Ne 1s photoelectrons and high kinetic energy ETMD electrons by inelastic scattering.
2. Production of additional low kinetic energy electrons by inelastic scattering of Auger electrons, or of Ne valence shell or Kr photoelectrons.

(1.) The onset of inelastic collisions with Ne atoms (the major fraction of atoms in the cluster) is expected at an excess energy of 17.6 eV[7]. Therefore, losses of Ne 1s photoelectrons at 888 eV photon energy and of high energy ETMD electrons due to inelastic scattering cannot be ruled out. For scattering of the ETMD electrons, at both photon energies probed this would lead to a slight downshift of the ETMD spectrum we observe. Scattering of the photoelectron, only possible at 888 eV, in consequence would lead to an overestimation of the number of ETMD electrons, as some photoelectrons are registered with an energy in the low kinetic energy band of secondaries. In pure Ne clusters of 70 and 150 mean size, a loss factor < 6 % due to this channel has been found at an excess energy of about 23 eV (the lowest that was measured), with a trend to drop towards the threshold of the channel[7]. We believe this figure to further scale down by a factor of at least two since our cluster size is much smaller. The potential losses are therefore much smaller than other sources of error in our data; also a propensity of low kinetic energy electron at the expense of Ne 1s electrons at 888 eV compared to 878 eV is not observed. Since Kr is a minority species in our clusters, we think it is justified to neglect scattering on Kr as well. These inelastic processes take place below the respective thresholds for electron impact ionization, therefore no free electrons in addition to the one being scattered may be produced.

(2.) Electron impact ionization due to intracluster inelastic scattering of high kinetic energy electrons (Auger electrons, photoelectrons from the Kr n = 3 shells, etc.) can potentially produce low kinetic energy electrons in the range that we interpret as being due ETMD. For processes driven by Kr-related electrons, this contribution should be eliminated by our background subtraction. This leaves us with the Auger electrons emitted from filling the Ne 1s core hole to consider. We believe the factor given above for inelastic photoelectron scattering (less than 3 %) is clearly an upper limit in the latter case as well, since the respective cross sections above a maximum at about 30 eV kinetic energy drop towards higher energies again[7].

Supplementary References

1. Veigele, WM. J. Photon cross sections from 0.1 keV to 1 MeV for elements Z = 1 to Z =94. *Atomic Data Tables* **5,** 51–111 (1973).
2. Suzuki, I. H. & Saito, N. Photoabsorption cross-section for Kr in the sub-keV energy region. *J. Electron Spectrosc. Relat. Phenom.* **123,** 239–245 (2002).
3. Saito, N. *et al.* Evidence of radiative charge transfer in argon dimers. *Chem. Phys. Lett.* **441,** 16–19 (2007).
4. Stoychev, S. D., Kuleff, A. I., Tarantelli, F. & Cederbaum, L. S. On the doubly ionized states of Ar_2 and their intra- and interatomic decay to Ar_2^{3+}. *J. Chem. Phys.* **128,** 1–10 (2008).
5. Fasshauer, E., Förstel, M., Mucke, M., Arion, T. & Hergenhahn, U. Theoretical and experimental investigation of Electron Transfer Mediated Decay in ArKr clusters. *Chem. Phys.* (2016). doi:10.1016/j.chemphys.2016.09.006
6. Stumpf, V., Kolorenč, P., Gokhberg, K. & Cederbaum, L. S. Efficient Pathway to Neutralization of Multiply Charged Ions Produced in Auger Processes. *Phys. Rev. Lett.* **110,** 258302 (2013).
7. Hergenhahn, U. *et al.* Observation of excitonic satellites in the photoelectron spectra of Ne and Ar clusters. *Chem. Phys. Lett.* **351,** 235–241 (2002).
